# Neuroenhancement and mental health in students from four faculties – a cross-sectional questionnaire study 

**DOI:** 10.3205/zma001664

**Published:** 2024-02-15

**Authors:** Maurice Hajduk, Elena Tiedemann, Marcel Romanos, Anne Simmenroth

**Affiliations:** 1 University Medical Centre Würzburg, Institute of General Practice, Würzburg, Germany; 2 University Medical Centre Würzburg, Clinic and Polyclinic for Child and Adolescent Psychiatry, Psychosomatics and Psychotherapy, Würzburg, Germany

**Keywords:** medical student, neuroenhancement, cognitive enhancement, brain doping, smoking, alcohol

## Abstract

**Background:**

Students face great challenges at the beginning of and during their studies. Competitive experience, exam anxiety, and especially the new performance requirements often cause test anxiety and stressful experiences. The extent of substance use in terms of neuroenhancement (NE) is unclear. Evidence shows associations between NE, increased stress levels, and mental health.

**Objectives:**

We aim to determine the prevalence of NE and alcohol and tobacco use among college students. We also investigate the associations between NE and ADHD, anxiety, depression, and stress experience.

**Methods:**

In spring 2021, an anonymous online cross-sectional survey was conducted among students of medicine, dentistry, business economics, and business informatics in Würzburg. The survey included the instruments ASRS (ADHD), PSS-10 (stress), PHQ-4 (depression and anxiety), and AUDIT-C (alcohol comsumption), as well as questions about consumption patterns, prior knowledge, and reasons for NE.

**Results:**

Of the 5564 students who were invited to participate, 1010 completed the questionnaire (18.2%). Of these, 12.4% indicated NE for the studied period. NE was used in particular during preparations for exams, to enhance performance, and/or to regulate emotions, most commonly through caffeine tablets, cannabis, and methylphenidate. NE was associated with risky use of alcohol or tobacco, and to a lesser extent with ADHD symptoms and stress experience.

Conclusions: Students are at risk of substance abuse and NE. Effective stress management and prevention approaches as well as low-threshold services are needed to identify and support students with risk profiles.

## Zusammenfassung

**Hintergrund:**

Während des Studiums stehen Studierende vor großen Herausforderungen: Leistungsanforderungen seitens der Hochschulen, Konkurrenzerleben, Prüfungsangst und die neu gewonnene Selbstständigkeit können zu Stresserleben, Angst und Depressivität führen. Die vorliegende Studie untersucht, inwieweit psychisch stärker belastete Studierende häufiger Neuroenhancement (NE) nutzen.

**Zielsetzung:**

Ermittlung der Häufigkeit von NE, Alkohol- und Tabakkonsum unter Studierenden sowie Untersuchung der Zusammenhänge zwischen NE und ADHS, Angst, Depressivität und Stresserleben.

**Methodik:**

Im Frühjahr 2021 wurde eine anonyme Online-Querschnittsbefragung bei Studierenden der Medizin, Zahnmedizin, Wirtschaftswissenschaften und -Informatik an Würzburger Hochschulen durchgeführt. Der Survey umfasste die Instrumente ASRS (ADHS), PSS-10 (Stress), PHQ-4 (Depressions- und Angstsymptome) und AUDIT-C (riskanter Alkoholkonsum), sowie Fragen zu Konsumverhalten, Vorwissen und Gründen für NE.

**Ergebnisse:**

1010 von 5564 Studierenden nahmen am Survey teil (18.2%). Davon gaben 12.4% NE für den Zeitraum des Studiums an. NE wurde insbesondere während der Prüfungsvorbereitung zur Leistungssteigerung und/oder zur emotionalen Regulation eingesetzt, am häufigsten durch Koffeintabletten, Cannabis und Methylphenidat. NE war assoziiert mit riskantem Alkohol- bzw. Tabakkonsum, im geringeren Ausmaß mit ADHS-Symptomen und Stresserleben.

Schlussfolgerung: Bei Studierenden besteht ein Risiko für Substanzmissbrauch und NE. Wirksame Stressbewältigungs- und Präventionsansätze sowie niederschwellige Hilfsangebote sind erforderlich, um Studierende mit Risikoprofil zu erkennen und zu unterstützen.

## 1. Background

Students face major challenges at the beginning of their studies: Many leave home for the first time, reorient themselves socially, and may have to deal with exam anxiety and stress. In order to enable themselves to face these challenges, students consume alcohol, nicotine, and illegal (e.g. cannabis, speed, or cocaine) and prescription substances (e.g. methylphenidate, modafinil, or beta-blockers) [1], [2] to learn or relax more efficiently [3]. We refer below with the term “neuroenhancement” (NE) to the use of substances with the aim of coping with the challenges of studying. The substances in these cases are not prescribed on the basis of a medical diagnosis. Depending on the substances that were investigated, the prevalence of NE in European student populations ranges from <1% to 22.5% [4], [5], [6], [7]. 

While NE in healthy individuals is seen as an abusive substance use to enhance personal performance, there is increasing evidence of a link between mental disorders and NE. For example, NE with methylphenidate has been described as a self-medication strategy in people with undiagnosed symptoms of attention-deficit hyperactivity disorder (ADHD) [6], [8], [9]. Further findings suggest an association with increased stress levels [10], [11] and anxiety [6], [11], [12], whereas the relation with depression is unclear [8], [11], [12]. Increased alcohol consumption and smoking also appear to be associated with NE [2], [12], [13], and it may indicate a generally increased propensity to substance use [13].

Because consistent data on NE among students are lacking, especially among medical students, we conducted an anonymous survey to determine the prevalence of NE, alcohol, and tobacco use. In addition, we investigated the relation between NE and mental health, in particular, symptoms of ADHD, anxiety, depression, and stress.

## 2. Methods

### 2.1. Study design and sample

In the winter semester 2020/21, we conducted an anonymous online cross-sectional survey to which we electronically invited a total of 5564 students. They were students from the Faculty of Medicine and Dentistry at the Julius-Maximilians-Universität Würzburg (n=2764) and the Faculty of Business Informatics and Economics at the Würzburg/Schweinfurt University of Applied Sciences (n=2800). The students at the university of applied sciences were selected to compensate for the high proportion of women studying medicine.

### 2.2. Questionnaire

The questionnaire consisted of 53 closed questions. No German-language validated instruments are available for the assessment of NE. The questions on NE were therefore formulated based on Middendorf et al. [14]. The questionnaire contained a brief definition of NE and a list of substances we categorise as NE in this study (methylphenidate, modafinil, dexamphetamine, atomoxetine, beta-blockers, antidepressants, anti-dementia drugs, caffeine tablets, cocaine, MDMA (ecstasy), amphetamines (speed), and cannabis). In addition, knowledge about NE and its use by fellow students, the prescription of one of the substances mentioned, and personal NE experiences during the study period were each asked dichotomously (yes/no). If the answer was yes, all substances that were used could be selected, and the motive for taking each substance could be specified (e.g. to increase concentration or to calm down). The deviation from a prescribed medication was recorded in a separate question (yes/no). The questionnaire concluded with a closed item to record tobacco consumption. The students were classified as smokers if they reported “daily” or “occasional” smoking.

The following validated screening instruments were used:

- ADHD: ADHD Self-Report Scale V1.1 Screener (ASRS-V1 [15])
- Stress: Perceived Stress Scale (PSS-10 [16])
- Depression and anxiety symptoms: Patient Health Questionnaire-4 consisting of GAD-2 and PHQ-2 (PHQ-4 [17])
- Risky alcohol consumption: Alcohol Use Disorders Identification Test-Consumption (AUDIT-C [18])

### 2.3. Data collection

The questionnaire was created electronically using EvaSys^®^ 7.1. A direct link was distributed by email by the responsible deans of studies and via the student councils. It was aimed at all students of human medicine and dentistry (university) and at students of business informatics and economics (university of applied sciences). The link was active between 20 January 2021 and 9 March 2021. Students were reminded of the survey at least once via WhatsApp or Facebook groups. To increase the response rate, there was the opportunity to take part in a prize draw for cinema vouchers via a separate window after completing the questionnaire. The survey was also advertised through notices in the university hospital. The data collection was completely anonymous.

### 2.4. Ethics, data management, and data protection

A positive vote from the Ethics Committee of the Medical Faculty of the University of Würzburg (No. 2020050502) has been received. The security, anonymity, and legal compliance of the questionnaire and data management were confirmed by the data protection officer of the University Hospital of Würzburg.

### 2.5. Data analysis

The characteristics of the participants were analysed descriptively by subject with regard to age, semester of study, and NE, provided that this information was available on the evaluated variable (no case exclusion). For data protection regulations, it was not possible to survey the gender of the students because individuals might be identified in small subgroups (e.g. minor subject or third-gender category). 

If cut-off values are established for the screening instruments, the results were evaluated using these cut-off values:

- ADHD: four or more positive screening items (item 1-3: ≥2 points, item 4-6: ≥3 points) in the ASRS-V1.1 or current ADHD diagnosis
- Depression symptoms: PHQ-2: ≥3 points
- Anxiety symptoms: GAD-2: ≥3 points
- Risky alcohol consumption: AUDIT-C: ≥4 points

Correlations were calculated using the Pearson correlation coefficient. To investigate relations between screening results and NE, a binomial logistic regression model was calculated for each instrument. Only data sets that were complete for ASRS, PSS-10, PHQ-4, AUDIT-C, and tobacco use were used (listwise case exclusion). All data were analysed with SPSS version 26.0, and p-values <0.05 were considered statistically significant.

## 3. Results

### 3.1. Sample description

Of the 5564 students we surveyed, 1010 completed the questionnaire (response rate: 18.2%). The respective proportion of respondents is shown in table 1 (Tab. 1). The largest group (n=594, 61.9%) consisted of 21-25-year-old students (born 1996-2000). They were followed in descending order of frequency by those born 1991-1995 (n=225, 23.5%), >2001 (n=82, 8.6%), and <1990 (n=58, 6%). The age distribution varied only slightly across the departments. The number of students in the first to fourth semester (n=394, 39%) did not differ significantly from those in the fifth to eighth semester (n=406, 40.2%). At the time of the survey, 20.8% of the participants (n=210) had studied for more than eight semesters.

### 3.2. Neuroenhancement

In the 1010 completed questionnaires, 125 students affirmed that they had practiced NE during their studies. This places the prevalence of NE during studies at 12.4%. The prevalence of NE during studies, knowledge about NE, and the proportion of fellow students who are known to use substances for NE are shown in table 2 (Tab. 2) by subject.

One hundred and twenty-four students provided information on the regularity of NE: About half of the students (n=61) denied regular use in the last three months. Almost daily use was reported by 11.3% (n=14), weekly use by 15.3% (n=19), and monthly use by 9.7% (n=12) of the students. 

Almost half of the students with NE experience reported NE in the last 30 days (n=57). For n=25 (20.3%), NE had been used between 30 days and a year ago, for a third, at least a year had passed since the last use. The most common reason given for NE was exam preparation (n=102, 10.1% of the total sample; 81.6% of the users); NE was less common during the semester (n=50, 5% of the total sample; 40% of the users).

A doctor’s prescription for one of the substances in the questionnaire had been obtained by 4.7% (n=47) of the participants. Of these, 17.0% (n=8) had deviated from the prescribed dose for NE purposes. When we included deviations from the prescribed dose as NE, the prevalence of NE increased from 12.4% to 12.7% (n=128).

**Figure 1:**
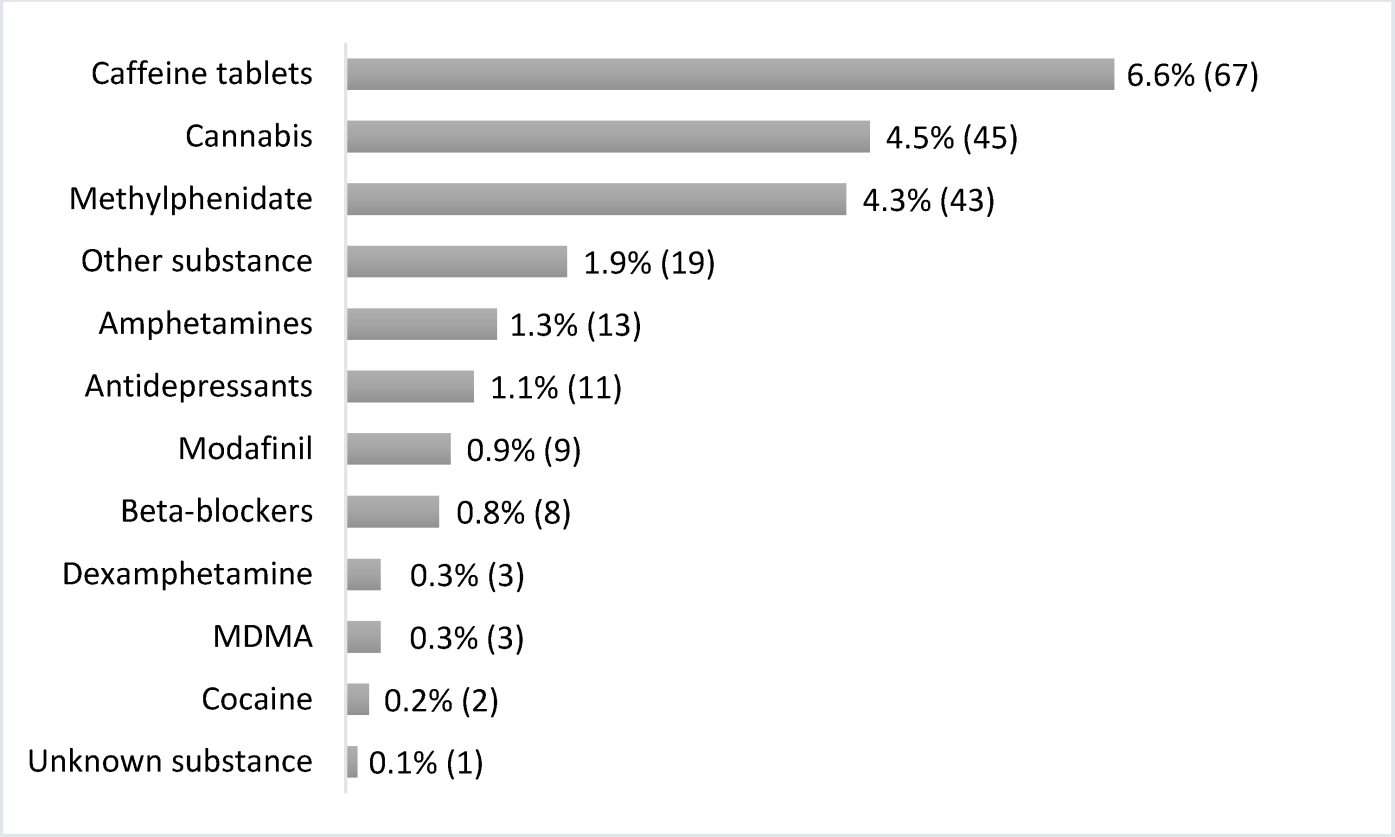
Substances used for NE. The percentages are given in relation to the total sample. The frequencies are shown in brackets.

Caffeine tablets, cannabis, and methylphenidate were most frequently used for NE (see figure 1 (Fig. 1)). Caffeine tablets, methylphenidate, modafinil, and amphetamines were mainly used to improve concentration, alertness, endurance, or performance. Cannabis, beta-blockers, and antidepressants, on the other hand, were used to improve inner calm and balance.

A diagnosis of ADHD was reported by 34 people (3.5%). Most of the diagnoses were made in childhood (n=24, 70.6%), and significantly fewer in adolescence and adulthood (n=5, 14.7% each). Approximately one-third of these individuals were taking the prescribed dose of ADHD medication at the time of the study (n=12, 35.3%). These cases were not classified as NE.

Table 3 (Tab. 3) provides an overview of the positive screenings for ADHD, depression, anxiety, risky alcohol use, and smoking by study. In the overall sample, screening was most frequently positive for risky alcohol use (n=366, 38.2%) and anxiety (n=330, 32.7%). Smoking was rare overall (n=110, 11%). 

The mean score for stress in the PSS-10 was M=28.6 (SD=6.8, n=989). The highest values were found among dental students (M=31.7, SD=7.2, n=74), and the lowest values among human medicine students (M=28.0, SD=6.6, n=498). 

For the following analyses, only fully completed screening instruments were considered. This reduced the number of cases to 912 (90.3% of the initial sample). ADHD, stress, anxiety, and depression all correlated significantly with each other, with anxiety and stress showing the highest correlation (r=.63). Depression was also significantly related to smoking and risky alcohol consumption (r=.07), and smoking was related to risky alcohol consumption (r=.16). The correlations are shown in table 4 (Tab. 4). The correlations between the predictor variables were low overall (r<.70). This indicates that multi-collinearity is not a confounding factor for the following logistic regression analysis.

The results of a binomial regression model for each variable individually and for the combination of all variables are shown in table 5 (Tab. 5). The overall binomial regression model was statistically significant, χ^2^(6)=28.40, p<0.001. The odds of NE were 2.49 times higher in smokers than in non-smokers (AOR 2.49, 95% CI 1.50-4.11, p<0.001). Risky alcohol consumption and NE are also positively associated (AOR 1.56, 95% CI 1.05-2.32).

## 4. Discussion

In this study, NE was examined in a large cohort of students from four disciplines. The sample consisted of an equal number of students from the lower and higher semesters. This means that the preclinical and clinical stages of medical studies are represented in a balanced ratio. Students reported a prevalence of NE of 12.4% during their studies. NE was mainly used in times of exam preparation, with caffeine tablets being consumed most frequently. Authors from Germany and Switzerland also reported similarly high prevalence rates when caffeine tablets are counted as NE [3], [19]. Experience with and knowledge of NE in our study agree well with the existing literature [20], [21], [22], with medical students showing higher knowledge of NE [5]. The symptom levels of the screenings for ADHD, anxiety, and depression were slightly higher than in the German general population [23], [24]. Even though validated instruments were used, the screening procedure does not yet correspond to a clinical diagnosis, so that these results must be interpreted with caution.

Anxiety, depression, and stress symptoms increase in students during exam periods [25]. The same applies to the period during the COVID-19 pandemic [26]: The data collection in winter 2021 coincided with a lockdown phase, when almost all teaching was made online. Another study from the same period found positive anxiety or depression screening in one-fifth of the medical students that were surveyed, which is comparable to our results [27]. Stress levels were even higher in our sample than in other German students during the pandemic [28].

NE was positively associated with symptoms of ADHD. ADHD is an important risk factor for substance abuse, and our finding of higher illicit or non-prescribed substance use in terms of NE in individuals with ADHD symptoms is supported by the literature [24]. 

Links of anxiety, depression, and stress to SD have repeatedly been reported [2], [11], [29]. In our study, stress was only weakly but significantly associated with NE, with stress showing high correlations with anxiety and depression. Although we did not have detailed insight into the specific motivation for NE, the associations may suggest that NE is used as a coping strategy to reduce stress by increasing the cognitive performance and attenuating negative emotions. This interpretation could apply to exam phases in particular. On the other hand, stress, depression, and anxiety symptoms could also be a consequence of substance use. This cannot be assessed in a cross-sectional study, however.

In a model with all screening instruments, only correlations of SD with risky alcohol consumption and tobacco use were found. This is similar to the results in other studies [12], [13]. The comparison of the mean values of perceived stress between the groups with and without risky alcohol consumption or between smokers and non-smokers showed no significant differences. This suggests that people who consume alcohol or nicotine are generally more prone to riskier health behaviours.

### 4.1. Limitations and strengths

Our response rate was somewhat lower at 18% than in similar studies (25% [2] and 22% [3]). The relatively low response rates could be related to the coincidence with examination times (end of semester). The temporal proximity to the second lockdown of the COVID-19 pandemic is a strength, but also a limitation. The semester was largely held online, and social interaction was reduced to a minimum since December 2020. This may limit comparability with studies from before the pandemic. However, our study provides valuable insights into the consumption behaviour regarding NE, alcohol, and tobacco, as well as into the mental health of students during the pandemic. Nevertheless, the results must be interpreted with caution because some of the predictors were strongly correlated, but none higher than 0.7.

Gender was not allowed to be collected in order to maintain anonymity. Because 72% of the medical students in Würzburg are female, and because men are more likely to engage in risky health behaviours such as alcohol consumption and smoking [30], [31], we selected study programmes with a higher proportion of male students (business informatics and business sciences). However, the medical students still represented the largest subgroup, which may have led to an underestimation of the prevalence of NE because medical students consume less. The start page of the survey may have had a strong effect on the selection of participants by making the topic of NE salient, which could have led to an overestimation of NE. Furthermore, we did not control for multiple participation. 

The strengths of our survey are the large sample size, especially in view of the fact that this study is the first to address the topic of NE at Würzburg University. Furthermore, good data quality was achieved through the use of established screening instruments.

### 4.2. Conclusion for practice

- NE is a common phenomenon among students. It is associated with mental health problems, although the underlying causal and temporal relations remain unclear.
- NE can be interpreted as a marker for a need for psychosocial support for students.
- Students who are under severe psychological pressure or are at risk could benefit from stress-management programmes, among other things. The programmes should aim to reduce NE by teaching alternative coping strategies.
- The continuation of research on NE in Würzburg after the pandemic and with an expansion of the sample to other specialist areas is desirable.
- A longitudinal study design would offer the opportunity to investigate changes in NE and mental health during the course of studies.

## Notes

### Authors’ ORCIDs

- Maurice Hajduk: 0009-0005-4538-5316
- Elena Tiedemann: 0009-0003-2393-6020
- Marcel Romanos: 0000-0001-7628-8299
- Anne Simmenroth: 0000-0002-3521-1225

### First authorship

The authors Maurice Hajduk and Elena Tiedemann share the first authorship.

## Competing interests

The authors declare that they have no competing interests. 

## Figures and Tables

**Table 1:**
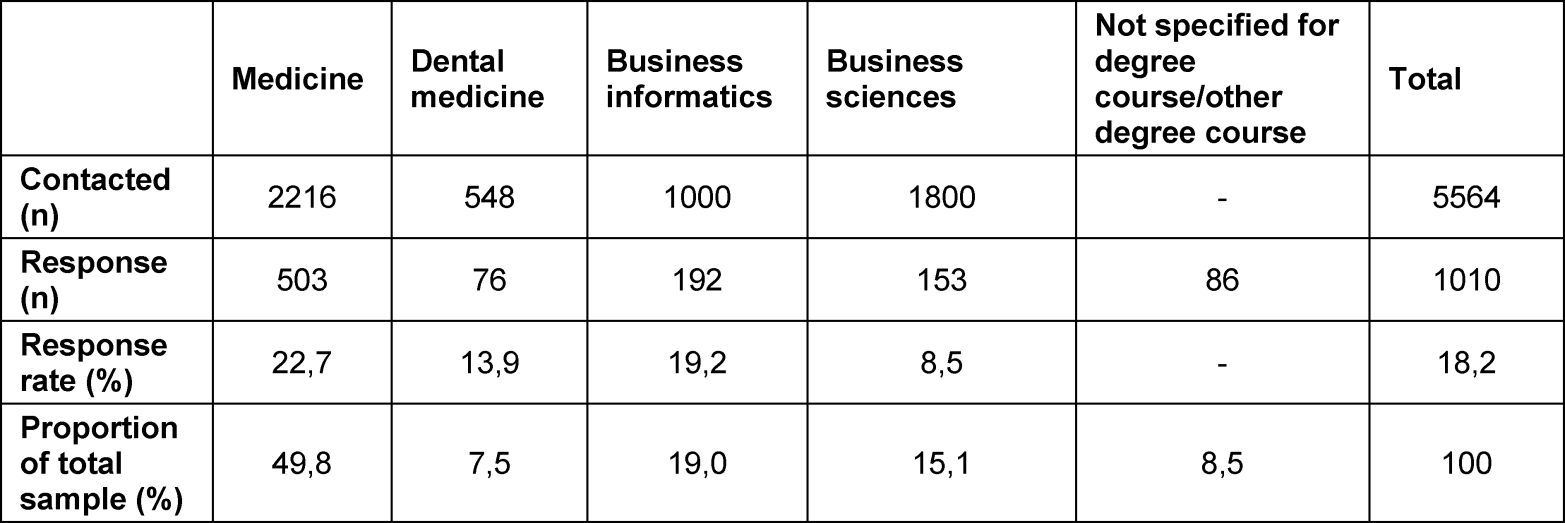
Response rate and proportion of total sample by study programme

**Table 2:**
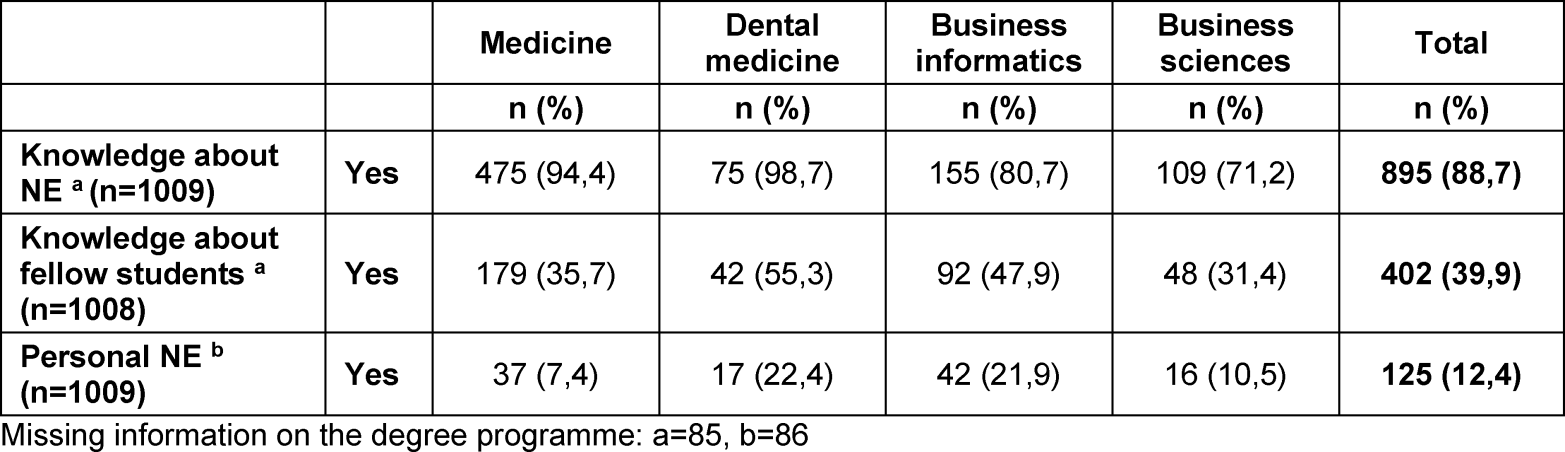
Knowledge, awareness of consumption by fellow students, and personal NE by study programme

**Table 3:**
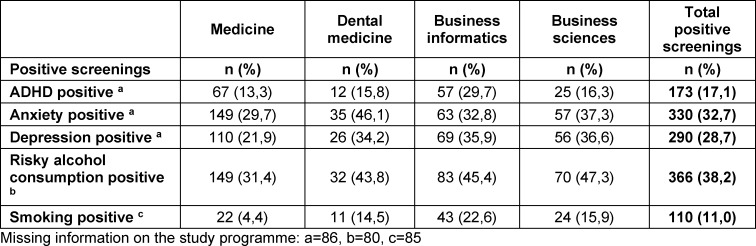
Positive screenings for ADHD, depression, anxiety, risky alcohol consumption, and smoking by study programme

**Table 4:**
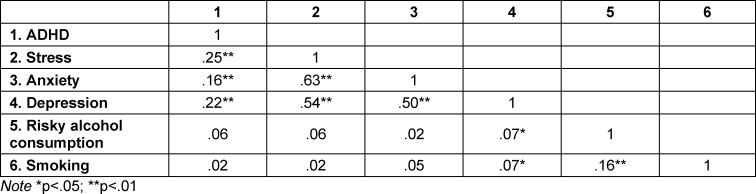
Correlation of the screening instruments (n=912)

**Table 5:**
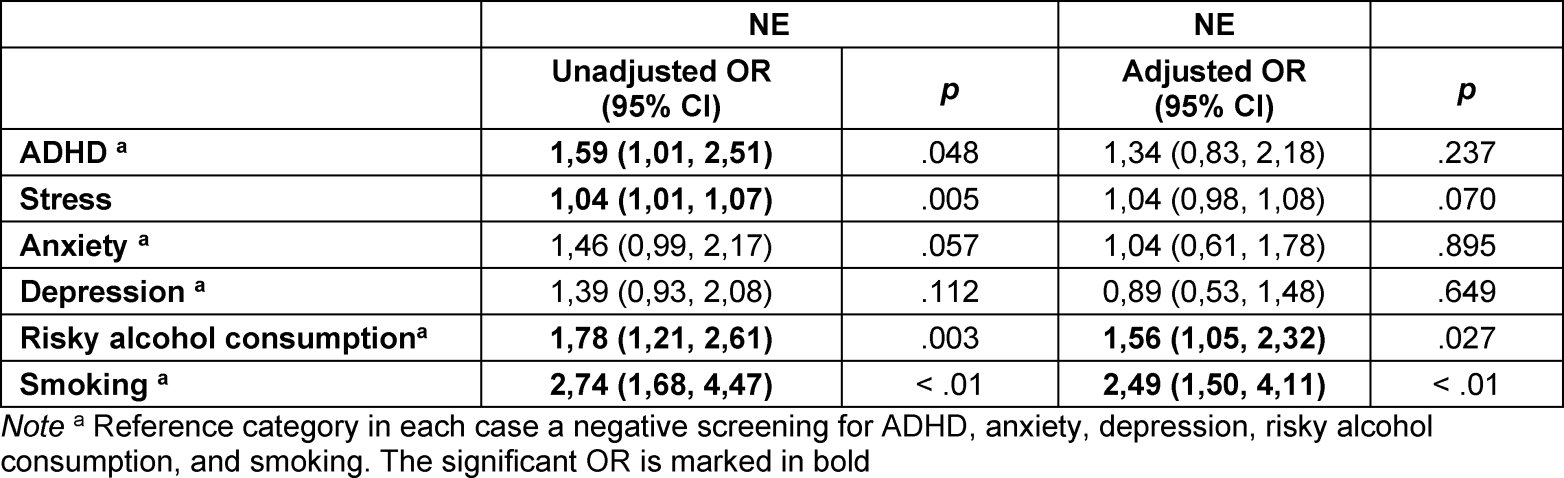
Comparison of the unadjusted and adjusted odds ratios (OR) of the independent variables ADHD, stress, anxiety, depression, risky alcohol consumption, and smoking in relation to the dependent variable NE (N=912)
